# Pentraxin 3 as a Modulator of miRNAs and Extracellular Vesicles Release in Triple-Negative Breast Cancer Cells

**DOI:** 10.3390/biomedicines14010014

**Published:** 2025-12-20

**Authors:** Diogo Gomes da Costa, Fábio Ribeiro Queiroz, Flávia Santiago de Oliveira, Angelo Borges de Melo Neto, Marina Malheiros Araújo Silvestrini, Ludmila Rodrigues Pinto Ferreira, Isabela Aurora Rodrigues, Virgínia Mendes Russo Vallejos, Adriana Oliveira Costa, Frédéric Frézard, Jorge Gomes Goulart Ferreira, Matheus de Souza Gomes, Andréa Teixeira-Carvalho, Paulo Guilherme de Oliveira Salles, Letícia da Conceição Braga, Adriana Abalen Martins Dias

**Affiliations:** 1 Department of Genetics, Ecology and Evolution, Institute of Biological Sciences, Federal University of Minas Gerais, Belo Horizonte 31270-901, Brazil; 2 Translational Oncology Research Laboratory, Teaching, Research and Innovation Center, Mário Penna Institute, Belo Horizonte 30380-420, Brazil; 3 Patos de Minas Campus, Federal University of Uberlândia, Patos de Minas 38701-002, Brazil; 4 Oswaldo Cruz Foundation (Fiocruz Minas), René Rachou Institute, Belo Horizonte 30190-002, Brazil; 5 Department of Morphology, Institute of Biological Sciences, Federal University of Minas Gerais, Belo Horizonte 31270-901, Brazil; 6 Department of Clinical and Toxicological Analyses, Faculty of Pharmacy, Federal University of Minas Gerais, Belo Horizonte 31270-901, Brazil; 7 Department of Physiology and Biophysics, Institute of Biological Sciences, Federal University of Minas Gerais, Belo Horizonte 31270-901, Brazil

**Keywords:** Pentraxin 3, breast cancer, EVs, triple-negative breast cancer, TNBC

## Abstract

**Background/Objectives**: Breast cancer is the most prevalent tumor among women worldwide, with the triple-negative (TNBC) being the most aggressive and therapeutically resistant subtype. It is crucial to investigate new therapeutic targets for the treatment of TNBC. Pentraxin 3 (PTX3), an acute-phase protein, has a complex role in tumor progression, with its expression associated with disease severity. We investigated the role of recombinant human PTX3 (rhPTX3) in modulating microRNA (miRNA) expression and extracellular vesicle (EV) release in TNBC MDA-MB-231 cells. **Methods**: PTX3 gene expression was evaluated by RT-qPCR. The miRNA expression profile was determined by small RNA Next-Generation Sequencing (NGS). EV release was analyzed by nanoparticle tracking analysis (NTA), flow cytometry, and protein quantification. **Results**: rhPTX3 treatment significantly increased PTX3 gene expression in MDA-MB-231 cells. Furthermore, rhPTX3 altered the expression profile of 142 miRNAs, with 112 being upregulated and 30 downregulated. These differentially expressed miRNAs were predicted to have 12,894 potential targets, impacting 29 canonical pathways related to carcinogenesis. Key molecules for cancer progression were inhibited (IL6, IL4, CXCL8, CXCR4, CXCL12; ICAM1, CD44 and BCL2), and pro-apoptotic BAD was activated. While rhPTX3-treatment increased total EV release, it specifically reduced the percentage of the CD44+ EV subpopulation. **Conclusions**: Our data demonstrates that PTX3 modulates the miRNA expression profile and EV release dynamics, particularly by reducing the CD44+ EV population, which points to a tumor-suppressor role in this TNBC context. Given the limited therapeutic avenues for TNBC, our results suggest that PTX3 and its downstream molecular effects represent promising and previously unexplored potential therapeutic targets.

## 1. Introduction

Breast cancer is the leading cause of cancer-related mortality among women worldwide [1]. According to the World Health Organization (WHO), breast cancer accounted for approximately 670,000 new deaths globally in 2022, with only 0.5–1% of these cases occurring in men [2]. Among the molecular subtypes, triple-negative breast cancer (TNBC) is the most aggressive. It is characterized by an earlier onset in younger individuals [3], accounting for ∼10–20% of all breast cancer cases [4]. TNBC is associated with higher recurrence rates and lower overall survival compared to other subtypes [5]. Furthermore, the five-year survival rate for patients with TNBC is approximately 61.4%, in contrast to 86.5% in luminal subtypes and 79.1% in HER2-positive tumors [6]. Understanding of the molecular bases of this type of tumor is essential for the development of more effective therapeutic strategies and improved patient outcomes once the absence of estrogen receptors (ERs), progesterone receptors (PRs), and human epidermal growth factor receptor 2 (HER2) renders both endocrine therapy and conventional targeted therapy ineffective in the treatment of TNBC [7].

Pentraxin 3 (PTX3) is a phylogenetically conserved acute-phase protein and the best-characterized member of the long pentraxin family [8]. Its production mainly occurs in response to pro-inflammatory stimuli, such as cytokines tumor necrosis factor (TNFA) and interleukin-1 beta (IL1B) [9,10], toll-like receptor agonists, oxidized LDL, and other stimuli. Unlike C-reactive protein (CRP), produced exclusively in the liver in response to inflammation, PTX3 is locally synthesized by various cell types, including monocytes, macrophages, vascular endothelial cells, and fibroblasts [11]. Given that PTX3 is locally expressed at sites of inflammation, it serves as a more immediate and precise indicator of localized inflammatory responses compared to systemic markers as CRP [12].

Studies reported in the literature have demonstrated the potential role of PTX3 as a biomarker of severity in various types of cancer, such as prostate cancer, liposarcoma, glioma, and breast cancer [11,13]. However, its role in tumors is controversial, as it can trigger either oncogenic or tumor-suppressive activity depending on the context [11,13].

miRNAs are a class of small endogenous non-coding RNAs, ranging from 17 to 25 nucleotides in length, that regulate gene expression [14]. Alterations in miRNA expression profiles can reflect cancer progression. As a result, numerous studies have been conducted to assess this pattern as a prognostic factor in the identification of therapeutic targets in breast cancer [15,16,17].

Another critical component in cancer progression is the release of extracellular vesicles (EVs), membrane-bound structures of reduced dimensions that are secreted by cells in the extracellular environment. These vesicles play critical roles in intercellular communication, molecule transport, and the modulation of physiological and pathological processes [18]. Due to their characteristics, EVs influence tumor progression through cellular communication, RNA, DNA, and protein transport, thereby shaping the tumor microenvironment (TME) [19,20]. Furthermore, cancer cells use EVs to regulate essential processes, such as migration, chemoresistance, and metastasis [21,22].

Given the limited knowledge of the molecular pathways involved in TNBC and the promising role of PTX3 in various tumors, this study aims to investigate the role of PTX3 in regulating the miRNA expression profile and EV release in an in vitro TNBC model using the MDA-MB-231 cell line. Our results demonstrate that the protein PTX3 modulates the miRNA expression profile and EV release in these cells, indicating a potential function as a tumor suppressor in this cellular context.

## 2. Materials and Methods

### 2.1. Human Recombinant PTX3 (rhPTX3)

Human recombinant PTX3 (rhPTX3) was kindly supplied by Dr. Cecilia Garlanda (Humanitas Clinical Institute, Rozzano, Italy). The protein was expressed in Chinese Hamster Ovary (CHO) cells and subsequently purified through immunoaffinity chromatography under endotoxin-free conditions. Protein purity was verified by SDS-PAGE, and potential lipopolysaccharide (LPS) contamination was assessed using the Limulus Amebocyte Lysate (LAL) assay, which indicated levels below 124 pg LPS per mg of protein. No IL-6 induction was observed in monocytes exposed to the preparation.

### 2.2. Cell Culture

The triple-negative breast adenocarcinoma MDA-MB-231 (ATCC HTB-26) cell line was obtained from the American Type Culture Collection (ATCC) and cultured in RPMI-1640 (Gibco, Waltham, MA, USA) medium containing penicillin/streptomycin (Sigma-Aldrich, St. Louis, MO, USA) (100 U and 10 mg/mL, respectively) and 2 mM L-glutamine (Gibco, São Paulo, Brazil) and supplemented with 10% fetal bovine serum (FBS) (Gibco, São Paulo, Brazil) at 37 °C with 5% CO_2_. Cells (2.5 × 10^5^ cells per well) were seeded in triplicate in 24-well plates with 500 μL of culture medium and incubated in an atmosphere of 5% CO_2_ at 37 °C until the cell monolayer reached approximately 90% confluence. The culture medium was then replaced with a fresh medium without FBS (Gibco, São Paulo, Brazil), containing (or not) 10 μg/mL of rhPTX3, as indicated, and cells were incubated for 3, 6, 12, and 24 h. The same treatment conditions were applied to all subsequent assays. The absence of mycoplasma contamination was verified, as shown in Supplementary Figure S1.

### 2.3. PCR Analysis

After the treatment with rhPTX3, which was demonstrated to be sufficient according to previous studies from our research group, total RNA from MDA-MB-231 cells was extracted using the miRNeasy kit (Qiagen, Hilden, Germany), following the manufacturer’s instructions, as seen in Figure S2. For cDNA synthesis, 1 μg of total RNA from each sample was combined with 500 pg of oligo(dT) (Invitrogen, Carlsbad, CA, USA), and DEPC-treated water (1% diethyl pyrocarbonate, subsequently autoclaved) was added to a final volume of 5 μL. The obtained cDNAs were used as templates in RT-qPCR reactions employing specific primer pairs for the amplification of *PTX3* and the housekeeping gene *Glyceraldehyde-3-phosphate dehydrogenase (GAPDH)*, used as a normalization gene, as seen in Table S1. The SYBR Green Master Mix (Applied Biosystems, Waltham, MA, USA) and the ABI 7900 HT Real-Time PCR platform (Life Technologies, Grand Island, NY, USA) were used for RT-qPCR. Gene expression levels were evaluated following the relative quantification method described by Pfaffl et al. [23].

### 2.4. Construction of Small RNA Libraries

Total RNA (100 ng) extracted from MDA-MB-231 cells treated with rhPTX3 for 3 h, and its respective control (untreated), were used for library construction using the QIAseq^®^ miRNA Library kit (Qiagen, Germantown, MD, USA). The libraries were quantified using the Qubit 3.0^®^ (Invitrogen, Waltham, MA, USA), and their quality was assessed using the TapeStation 4150 (Agilent Technologies, Waldbronn, Germany). Finally, the library was denatured and sequenced on the NEXTseq 550 (Illumina, San Diego, CA, USA) using the High Output kit for 75 cycles (Illumina, San Diego, CA, USA). All procedures followed the manufacturer’s instructions. The sequencing data generated in this study have been deposited in the NCBI Sequence Read Archive (SRA) under the BioProject accession number PRJNA1310180.

#### Analysis of miRNA Sequencing Data

The obtained sequences were analyzed using the UNITAS software version 1.8.0 [24]. Adapter sequences and low-quality bases (<Q30) were removed, and sequences shorter than 15 nucleotides (nt) were discarded, as seen in Figure S3. The filtered RNAs were aligned to the human genome (GRCh38), and the miRBase v22 database was used to identify the miRNA sequences. Differentially expressed miRNAs (DEMs) were analyzed using the DESeq2 (v1.46.0) software package, and the prediction of target genes and their interaction networks was performed using the Ingenuity Pathway Analysis software (IPA, Qiagen, CA, USA, Version 150276282).

### 2.5. Isolation of EVs from Cell Culture Supernatant

An adapted version of the protocol proposed by Wang et al. [25] was applied. Cells were incubated for 24 h to obtain a sufficient quantity of EVs for subsequent analyses. During this incubation period, cells were treated with 10 μg/mL of rhPTX3. After incubation, the culture supernatant was collected and centrifuged at 700× *g* (5418 R, Eppendorf, Hamburg, Germany) for 10 min to remove cellular debris. Next, ultracentrifugation was performed at 4 °C for 4 h at 100,000× *g* (Sorvall, Ultra Pro80, Waltham, MA, USA). The supernatant was discarded, and EVs were resuspended in 150 µL of RPMI-1640 medium (pH 7.2), previously filtered through a 0.22 µm membrane (Millipore, Billerica, MA, USA). Samples were stored at −80 °C.

#### 2.5.1. Nanoparticle Tracking Analysis

The samples were diluted in PBS at a ratio of 1:100,000 and analyzed using the NanoSight LM14 (Malvern Instruments, Amesbury, UK) at high resolution for size distribution and concentration. Each sample reading was performed in technical quintuplicate, using 60 s videos at 25 frames per second (25 FPS) at a fixed temperature of 25 °C, with automatic detection. Images were captured by a charge-coupled device camera using the same camera gain. Analyses were conducted using the NTA 3.2 software (Malvern Instruments, Amesbury, UK).

#### 2.5.2. Flow Cytometry Analyses

Each EV sample (85 µL) was homogenized in 200 µL of annexin buffer (0.1 M Hepes/NaOH (pH 7.4), 1.4 M NaCl, 25 mM CaCl_2_). Subsequently, 4 µL of annexin V (FITC fluorochrome, BD Biosciences, San Jose, CA, USA) and 2 µL of anti-CD44 antibody (PE-Cy7 fluorochrome, BD Biosciences, San Jose, CA, USA) were added. The suspension was incubated at room temperature for 30 min. Finally, 300 µL of annexin buffer was added to each tube. The Cytoflex S flow cytometer (Beckman Coulter, Brea, CA, USA) was calibrated using fluorescent beads of defined sizes ranging from 100 to 900 nm. The standard acquisition of EVs was set at 120 s per sample. The results were analyzed using FlowJo v.10.1.8 software (Becton Dickinson, Ashland, OR, USA).

#### 2.5.3. Evaluation of Proteins Present in EVs

The protein concentrations of the EVs were estimated using the bicinchoninic acid (BCA) assay (Sigma, St. Louis, MO, USA), following the manufacturer’s recommendations. For the standard curve, the following Bovine Serum Albumin (BSA) concentrations were used: 0, 0.5, 5, 10, 20, and 30 µg/mL. Initially, 1 µL of each EV sample was diluted 1:10 in PBS and then added to a 96-well plate containing 10 µL of working reagent. Subsequently, the samples and standards were analyzed in a spectrophotometer with absorbance measured at 562 nm.

### 2.6. Statistical Analysis

Statistical analyses were performed using Prism 7 software (GraphPad Software, La Jolla, CA, USA) running on the Microsoft Windows 10 platform. For non-parametric data, comparisons between two groups were conducted using the Mann–Whitney test, while comparisons involving more than two groups were performed using the Kruskal–Wallis test. A *p*-value ≤ 0.05 was considered statistically significant. The significance of the association between each list of differentially expressed miRNA (DEM) targets and canonical pathways, as well as the relationships between molecules within the constructed networks, was assessed using Fisher’s exact test. *p*-values were adjusted by the Benjamini–Hochberg method, with adjusted *p*-values ≤ 0.05 considered significant to control the false discovery rate.

## 3. Results

### 3.1. rhPTX3 Treatment Increased PTX3 Gene Expression

**Figure 1:**
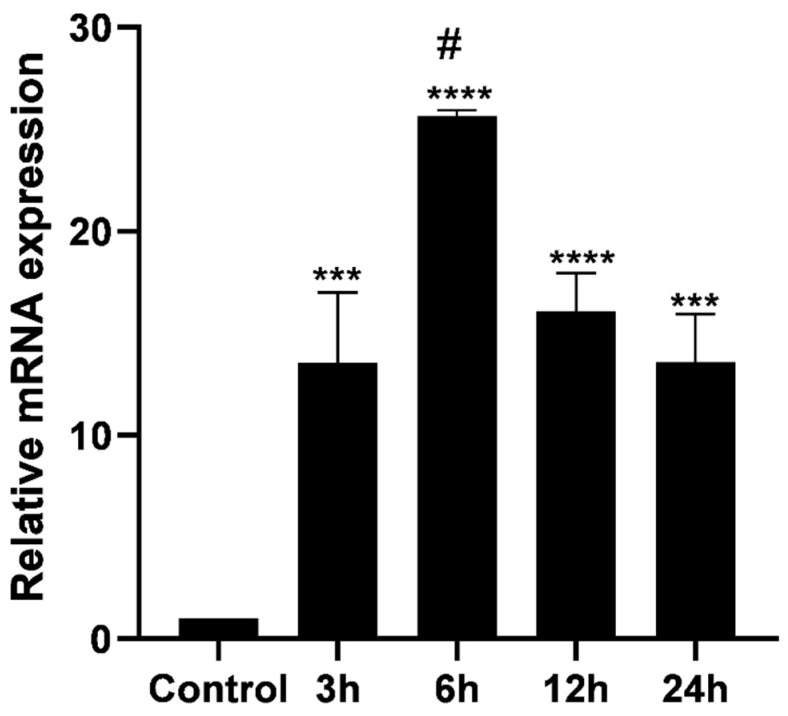
*PTX3* gene expression following treatment of MDA-MB-231 cells with rhPTX3. RT-qPCR analysis using specific primers for *PTX3* and *GAPDH* (reference gene) showed a relative increase in *PTX3* expression in all rhPTX3-treated groups compared to the control, with a significant peak in the 6 h group. Statistical analyses were performed using unpaired *t*-tests between control and treated groups (*** *p* < 0.001; **** *p* < 0.0001). Comparisons among all time points were performed using one-way ANOVA followed by Tukey’s post-test (# *p* < 0.01).

PTX3 mRNA transcription was increased in MDA-MB-231 cells treated with 10 μg/mL of the rhPTX3 at all time points (Figure 1), with the highest transcript accumulation at 6 h.

### 3.2. Transcriptome Analysis of Small RNAs

**Figure 2:**
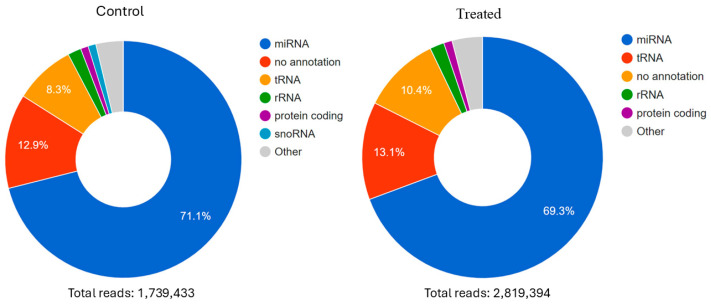
Profile of RNAs identified in the generated libraries. Small RNAs were mapped using miRBase. The representation of miRNAs was 71.1% in the control sample and 69.3% in the rhPTX3-treated sample. In addition to miRNAs, other small RNAs were also identified, including tRNA (transfer RNA), rRNA (ribosomal RNA), and snoRNAs (small nucleolar RNAs).

The total of filtered small RNA reads was 1,739,433 in the control group and 2,819,394 in the treated group, with approximately 71% representing miRNAs in the control group and 69.3% in the treated group (Figure 2). A total of 3703 different miRNAs were identified in the control group and 3800 in the treated group.

### 3.3. The Treatment with PTX3 Altered the miRNA Expression Profile in CMTN Cells

**Figure 3:**
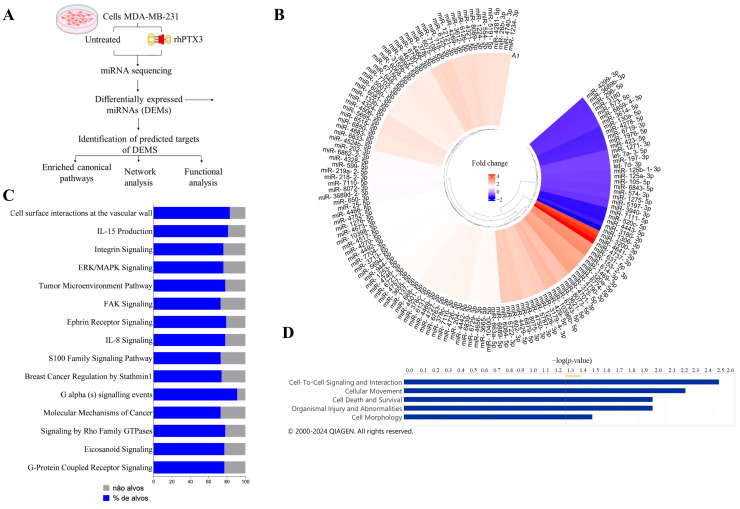
DEMs in rhPTX3-treated MDA-MB-231 cells in comparison with untreated cells. (**A**). Workflow illustrating the treatment of MDA-MB-231 cells with rhPTX3 through to in silico analyses. (**B**). The 3841 mapped miRNAs were subjected to differential expression analysis using DESeq2 software. As a criterion, miRNAs with a log2 fold change ≤−1 or ≥1 were considered DEMs. Thus, the 30 downregulated miRNAs are represented in blue in the heatmap, and the 112 upregulated miRNAs are shown in red. (**C**). Potential canonical pathways regulated by miRNAs following treatment with rhPTX3. IPA software (Version 150276282) was used to evaluate the canonical pathways regulated by the DEMs, which were filtered to obtain information specific to the MDA-MB-231 cell line. The bar graphs show the percentage of targets among the 15 main pathways related to breast cancer. The parentheses numbers represent the genes associated with each canonical pathway. The blue bars indicate the percentage of targets from the DEMs. The Benjamini–Hochberg statistical test was applied to adjust the *p*-value from Fisher’s exact test. (**D**). In IPA, the biological functions related to the DEMs identified in the miRNA sequencing were also analyzed. The Benjamini–Hochberg statistical analysis adjusted the *p*-value from Fisher’s exact test.

We identified 142 DEMs, with 30 miRNAs showing negative and 112 showing positive modulation of expression in response to the PTX3 treatment (Figure 3B). The DEM potential targets were predicted using IPA. Information was found for 122 DEMs, with 12,894 predicted targets. The analysis of enriched canonical pathways regulated by the DEMs identified a total of 29 enriched pathways using a specific filter to the MDA-MB-231 cell line, and 15 of them were selected as significantly relevant (Figure 3C). The potential biological functions impacted by these DEMs were also analyzed. The findings included cell signaling and cell-to-cell interaction, cell movement, cell survival, lesions, organ abnormalities, and cell morphology (Figure 3D).

### 3.4. Prediction of Breast Cancer-Related DEMs Networks

**Figure 4:**
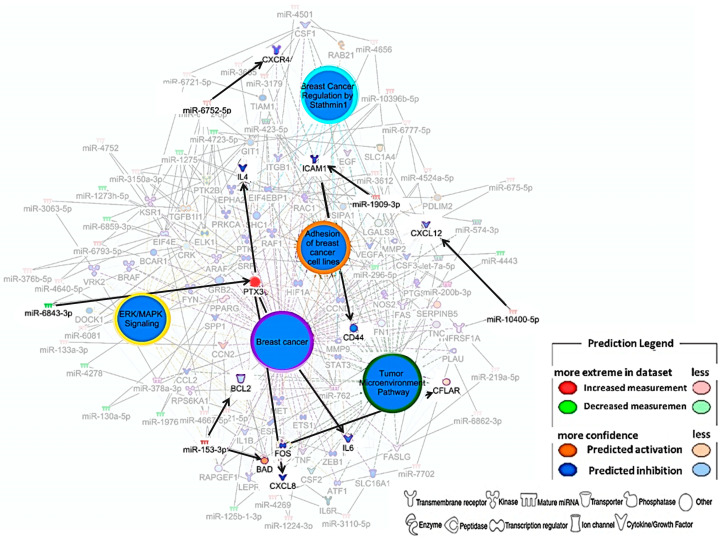
DEMs interaction network with their targets in breast cancer. The DEMs in red are positively regulated (darker shades represent more extreme regulation, while lighter shades indicate less extreme regulation). In contrast, the DEMs in green (darker shades for more extreme, lighter shades for less extreme) are negatively regulated. As shown in the image legend, each molecule has its symbol. Activated molecules are represented in orange (darker shades for more extreme, lighter shades for less extreme). In comparison, inhibited molecules are shown in blue (darker shades for more extreme, lighter shades for less extreme).

The predictive interaction network of miRNAs and their targets was constructed with a focus on key pathways in breast cancer, which were found to be inhibited—breast cancer regulation by Stathmin1, ERK/MAPK signaling, tumor microenvironment pathway, and adhesion of breast cancer cell lines. Among the various molecules potentially regulated in the network, we highlight the predicted activation of PTX3, BAD, and CFLAR, as well as the predicted inhibition of IL6, IL4, CXCR4, CXC12, CXCL8, ICAM1, CD44, and BCL2 (Figure 4).

Due to rhPTX3 treatment, PTX3 was highlighted in red in the network; however, its activation was predicted as a consequence of negative regulation by miR-6843-3p, which targets PTX3. This activation led to the inhibition of interleukins IL6 and IL4, as well as the chemokine CXCL8. The predicted inhibition of the chemokine receptor CXCR4 was associated with the positive regulation of miR-6752-5p, while CXC12 inhibition was attributed to miR-10400-5p activation. Additionally, the network predicted the inhibition of ICAM1 and CD44, molecules involved in cell–cell and cell–extracellular matrix interactions. The positive regulation of miR-1909-3p was associated with the inhibition of ICAM1, which, in turn, may have contributed to CD44 inhibition.

The results indicated a dual regulation of differentially expressed miRNAs in the context of apoptosis. The positive regulation of miR-153-3p was predicted to inhibit BCL2, an anti-apoptotic factor, which consequently led to the activation of the pro-apoptotic factor BAD. On the other hand, the predicted activation of the anti-apoptotic molecule CFLAR may be associated with the inhibition of the transcription factor FOS, suggesting a complex modulation of the balance between cell survival and apoptosis.

### 3.5. Impact of PTX3 Treatment on the EV Release

**Figure 5:**
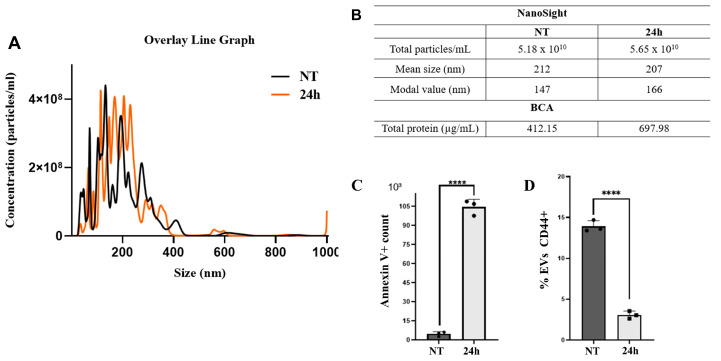
Size and concentration of particles isolated from the supernatant of MDA-MB-231. (**A**). In both groups, control (NT) and treated with rhPTX3 (24 h) the particle size predominantly ranged from 50 to 400 nm, with a small peak near 600 nm. Only the group treated with PTX3 exhibited a peak at 1000 nm. (**B**). The total particle counts measured by NanoSight and BCA showed higher values of total particles/mL and protein concentration in the rhPTX3-treated group (24 h) compared to the untreated group (NT). (**C**). Using flow cytometry, we observed that the rhPTX3-treated group (24 h) had 107,013 positive Annexin V events compared to 5327 events in the control group (NT), indicating an increase in EVs after treatment. (**D**). Furthermore, the percentage of CD44+ EVs released by MDA-MB-231 cells in the rhPTX3-treated group (24 h) was lower than in the control group (NT). A *t*-test was used to compare the control and treated groups with ****—*p* < 0.0001.

Nanoparticle tracking analysis (NTA) was performed to assess EV size and concentration (number of particles released per mL), as shown in Figures S4 and S5. The overlaid line graph revealed a distinct particle profile following treatment with rhPTX3 (Figure 5A). Considering particles ranging from 30 to 1000 nm, 5.16 × 10^10^ particles/mL were quantified in the control group and 5.64 × 10^10^ particles/mL in the treated group (Figure 5B). The particles exhibited a mean particle size of 203 nm and a modal value of 156 nm in the control group (NT), while in the group treated with rhPTX3 (24 h), they exhibited a mean particle size of 207 nm and a modal value of 141 nm were found. The control group presented a total protein concentration of 412.15 μg/mL, while the rhPTX3-treated group showed 697.88 μg/mL of protein content. Flow cytometry analyses significantly confirmed an increase in EV release in the group treated with rhPTX3 compared to the control (Figure 5C and Figure S6). Additionally, it revealed lower CD44+ EVs in the rhPTX3-treated group (Figure 5D).

## 4. Discussion

The role of PTX3 in neoplasia is extensively studied, with its expression varying considerably among different tumor types, disease stages, and cell lines [13]. PTX3 plays important roles in modulating the inflammatory response, remodeling the extracellular matrix, and regulating signaling pathways associated with cell proliferation and survival, which can directly influence tumor behavior. In some cases, PTX3 is associated with cancer progression, as observed in lung cancer, where it has been linked to increased proliferation, reduced apoptosis, and cisplatin resistance via activation of the Akt/NFKB pathways [26]. In gliomas, PTX3 is associated with poor prognosis and may favor tumor progression through the inhibition of autophagy [27]. Conversely, in other neoplasms, PTX3 acts as a tumor suppressor by antagonizing the Fibroblast Growth Factor (FGF) system. In fibrosarcoma, PTX3 inhibits FGF-dependent neovascularization and tumor growth [28]. Similarly, in bladder cancer, PTX3 expression is downregulated during progression to invasive forms, allowing for the hyperactivation of the FGF/FGFR axis, which contributes to drug resistance [29]. The dual role of PTX3 is also observed in breast cancer. PTX3 levels were elevated in patients with more advanced stages of the disease [30]. Additionally, PTX3 suppression reduced the cell proliferation rate and mitigated the effects of various cellular processes associated with metastasis, such as cell chemotaxis, migration, adhesion, and invasion [30]. On the other hand, PTX3 has been reported to attenuate breast cancer progression through its interaction with members of the FGF family, inhibiting angiogenesis and cell invasion [31]. This duality of PTX3 is expected due to its multifaceted activity, performing various functions and biological activities. This underscores the need to understand the specific contexts in which it is involved in breast cancer.

Choi et al. [32] demonstrated that MDA-MB-231 (TNBC) cells express higher levels of PTX3 protein compared to estrogen receptor-positive and progesterone receptor-positive human breast cancer cells, MCF-7. The more significant presence of PTX3 in the MDA-MB-231 lineage may be explained by the structural characteristics of these cells. Mohan et al. ([33]) showed that the MDA-MB-231 lineage has a higher representation of CD44+ cells than the MCF-7 lineage. PTX3 binds to CD44 in MDA-MB-231 cells, activating NFKB [34]. Given that NFKB is a key transcription factor for PTX3, it is plausible that PTX3 protein regulates its own gene expression in TNBC through a feedback mechanism involving this pathway. This hypothesis is supported by our findings, which demonstrate that rhPTX3 treatment significantly upregulates *PTX3* mRNA expression. In the present study, we demonstrate that the acute-phase protein PTX3 can alter the miRNA gene expression profile in TNBC cells. A total of 142 DEMs were identified, with a marked predominance of upregulated miRNAs (112) compared to downregulated ones (30). This pattern may be partially attributed to the previously reported role of PTX3 in activating NFKB in TNBC [34]. NFKB has been widely implicated in the transcriptional regulation of miRNAs, which can either promote or suppress tumor progression depending on the context [35]. However, further investigation is warranted to elucidate the mechanisms by which PTX3 modulates miRNA expression through NFKB activation.

Using IPA software, we demonstrated that the DEMs are related to five biological functions: cell signaling and cell–cell interaction, cell movement, cell survival, organ injury and abnormalities, and cell morphology. Furthermore, we identified 29 enriched canonical pathways, three of which (ERK/MAPK signaling, regulation of breast cancer by Stathmin1, and tumor microenvironment pathway) were highlighted in our interaction network.

In the interaction network, we observed that the miRNAs have important targets that may influence the regulation of key molecules in tumor progression. For example, the pro-apoptotic protein BAD appeared activated in our predictions. This protein is a crucial apoptotic activator in breast cancer cells; however, BAD protein expression is low in this type of tumor. The anti-apoptotic protein BCL2 appeared to be inhibited in our in silico analyses. Unlike BAD, BCL2 has anti-apoptotic activity [36]. Some chemokines were found regulated in our results, such as CXCL8, known for recruiting neutrophils to the TME, where N2-type neutrophils are associated with stimulating angiogenesis, contributing to tumor growth [37]. Additionally, we identified the inhibition of the chemokine receptor CXCR4 and its ligand CXCL12, both highly expressed in breast cancer and implicated in tumorigenic activities [38]. Moreover, the miRNAs also demonstrated the ability to inhibit molecules related to signaling and cell–cell interaction (ICAM and CD44) and inflammatory cytokines (IL6 and IL4).

Despite the growing number of studies investigating EVs released by cancer cells, and ongoing efforts by the scientific community to elucidate the underlying mechanisms of this process, the regulation and functional significance of EVs released by cancer cells remain incompletely understood [39]. In this study, we observed an increase in EVs release in the rhPTX3-treated group compared to the control group. This finding contrasts with previous studies that showed that *PTX3* knockout in MDA-MB-231 cells did not impact EVs release [22]. However, it is worth noting that the methodologies used in those studies differ from ours. While Wills et al. [22] chose to filter the cell supernatant to enrich smaller vesicles, which may have resulted in the exclusion of larger EVs, our study opted not to perform this filtration, allowing for the representation of EVs of all sizes.

We hypothesize that rhPTX3 treatment could increase EV release in the MDA-MB-231 lineage, activating the RHO family GTPase signaling pathway, a canonical pathway enriched by the observed DEMs. RHO family GTPases are known to regulate the biogenesis and release of EVs in cancer cells due to their crucial role in regulating cellular structure through actin cytoskeleton rearrangements [40]. However, we recognize that the limitations of our study require additional assays to determine whether this pathway is indeed mediating the rhPTX3-induced increase in EV release.

Additionally, our results indicated that, despite the rhPTX3 treatment increasing the EV release, it reduced the percentage of CD44+ EVs compared to the control. CD44 is a molecule often associated with chemoresistance and tumor aggressiveness, as evidenced in studies where treatment with Doxorubicin increased the release of CD44+ EVs, correlating with chemoresistance in breast cancer [21].

These observations suggest that PTX3 may play a role in regulating EVs, influencing both the quantity and composition of these vesicles, which may have important implications for tumor behavior and treatment response. The decrease in the release of CD44+ EVs by the rhPTX3 treatment group may contribute to a better understanding of the role of PTX3 in TNBC, and its potential to reduce CD44+ EVs should be explored in this type of tumor as a therapeutic target. It is essential to note that other EV-related factors, such as miRNA cargo, may also influence tumor development. For instance, EV-associated miRNAs are known to mediate intercellular communication in the tumor microenvironment, promoting metastasis, immune evasion, or drug resistance [41,42]. Although we did not characterize the miRNA content of EVs in this study, future studies should focus on unraveling the mechanisms by which rhPTX3 modulates the release and composition of EVs, including the sorting of specific miRNAs, and how this relates to tumor progression and chemotherapy resistance in TNBC.

Therefore, it is essential to highlight that these initial results regarding the potentially regulated miRNAs and their biological functions following treatment with rhPTX3 are exploratory. TNBC is a heterogeneous disease composed of distinct molecular subtypes. The MDA-MB-231 cell line is derived from the mesenchymal subtype and is widely used worldwide as an important model for studying TNBC. Our study provides relevant data obtained from this cell type and identifies candidate DEMs that warrant further validation through biological assays with different TNBC cell models, as well as in tumor samples from patients. Nonetheless, it is essential to emphasize the ability of PTX3 in our study to alter the expression of miRNAs that target oncogenic molecules enriched in TNBC and decrease the population of EVs known to increase chemoresistance in breast cancer. PTX3, miRNAs, and EVs are interrelated in various biological contexts in cancer. However, this is the first time the role of PTX3 in the presence of miRNAs and EVs in TNBC is being presented.

## 5. Conclusions

The acute-phase protein PTX3 modulates the miRNA expression profile and EV release in MDA-MB-231 human TNBC cells, potentially functioning as a tumor suppressor in this cellular context.

## Data Availability

The sequencing data generated in this study have been deposited in the NCBI Sequence Read Archive (SRA) under the BioProject accession number PRJNA1310180.

## Supplementary Materials

The following supporting information can be downloaded at https://www.mdpi.com/article/10.3390/biomedicines14010014/s1, Figure S1: Sequence of specific primers used for gene expression analysis by RT-qPCR; Figure S2: Analysis of total RNA integrity extracted from MDA-MB-231 cells treated or untreated with rhPTX3 using capillary electrophoresis (QIAxcel); Figure S3: Profile of Small RNAs Identified by UNITAS Software; Figure S4. Nanoparticle tracking analysis (NTA) of extracellular vesicles (EVs) isolated from the conditioned medium of MDA-MB-231 cells; Figure S5. NTA of EVs; Figure S6. Dot plots showing Annexin V positive cell populations; Table S1: Sequence of specific primers used for gene expression analysis by RT-qPCR.

## Abbreviations

The following abbreviations are used in this manuscript:

ATCC	American Type Culture Collection
BAD	Bcl-2-associated death promoter
BCA	Bicinchoninic acid
BCL2	B-cell lymphoma 2
BSA	Bovine Serum Albumin
CAPES	Coordenação de Aperfeiçoamento de Pessoal de Nível Superior
CRP	C-reactive protein
CXCL8	C-X-C motif chemokine ligand 8
CXCL12	C-X-C motif chemokine ligand 12
CXCR4	C-X-C motif chemokine receptor 4
DEMs	Differentially expressed miRNAs
ER	Estrogen receptors
ERK	Extracellular signal-regulated kinase
EV	Extracellular vesicle
FBS	Fetal bovine serum
FGF	Fibroblast Growth Factor
FPS	Frames per second
GAPDH	Glyceraldehyde-3-phosphate dehydrogenase
HER2	Human epidermal growth factor receptor 2
ICAM	Intercellular Adhesion Molecule
IL4	Interleukin 4
IL6	Interleukin 6
IPA	Ingenuity Pathway software
MAPK	Mitogen-activated protein kinase
miRNA	MicroRNA
MS	Ministério da Saúde
NGS	Next-Generation Sequencing
NFKB	Nuclear factor kappa-light-chain-enhancer of activated B cells
NT	Non-treated
