# Supplementary material for: Pentraxin 3 as a Modulator of miRNAs and Extracellular Vesicles Release in Triple-Negative Breast Cancer Cells
Source: Biomedicines. 2025 Dec 20;14(1):14. doi: 10.3390/biomedicines14010014 (PMC12838261; doi:10.3390/biomedicines14010014)
Supplement: Supplementary file 1 [file biomedicines-14-00014-s001.zip › biomedicines-3970371-supplementary.pdf]

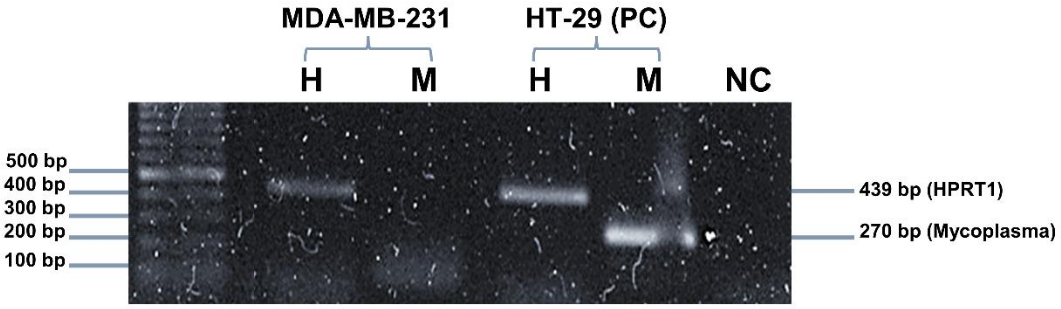

Figure S1. **Analysis of Mycoplasma contamination in the MDA-MB-231 cell line**. Genomic DNA from the human breast adenocarcinoma cell line MDA-MB-231 was extracted and used as a template in PCR reactions with primers specific for the gene encoding the 16S ribosomal RNA (rRNA) of *Mycoplasma* spp (M) as described by Van Kuppeveld et al (1994), and for the gene encoding hypoxanthine-guanine phosphoribosyltransferase 1 (H), used as a positive control for the presence of template DNA in the samples. NC: negative control (no DNA); PC: positive control (DNA from Mycoplasma-contaminated colorectal adenocarcinoma HT29 cells).

Table S1. Sequence of specific primers used for gene expression analysis by RT-qPCR.

Genes	Sequence	Amplicon (bp)	Efficiency
PTX3 *fw*	CGGCAGGTTGTGAAACAG	100	99,6%
PTX3 *rv*	GGCACTAAAAGACTCAAGCC		
GAPDH *fw*	CTCTCTGCTCCTCCTGTTC	100	93%
GAPDH *rv*	GATGATGACCCTTTTGGCTC		

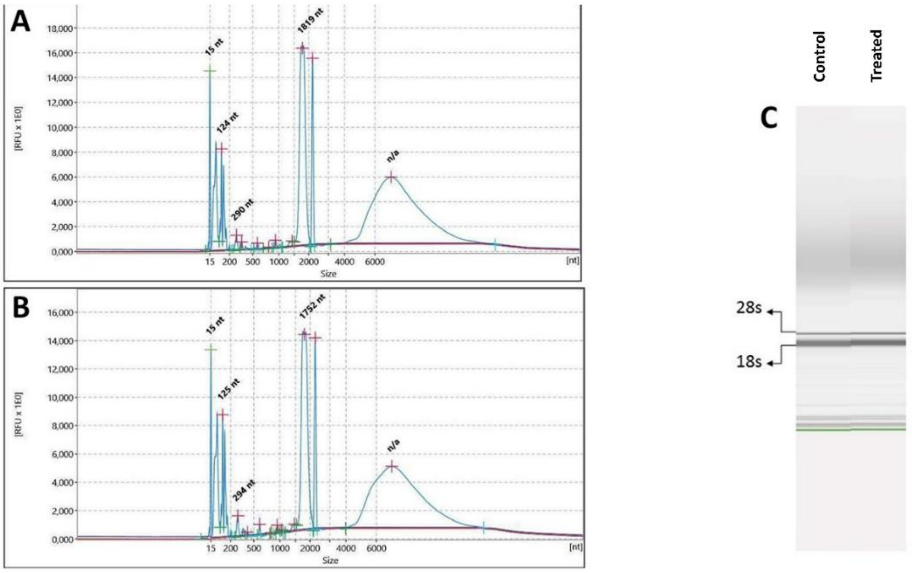

Figure S2: Analysis of total RNA integrity extracted from MDA-MB-231 cells treated or untreated with rhPTX3 using capillary electrophoresis (QIAxcel). Panel A shows the electropherogram of the control group. Panel B shows the electropherogram of the rhPTX3-treated group. Panel C shows gel image generated by QIAxcel analysis is shown in Panel C. The RNA quality score was 10 for the control group and 9.2 for the treated group.

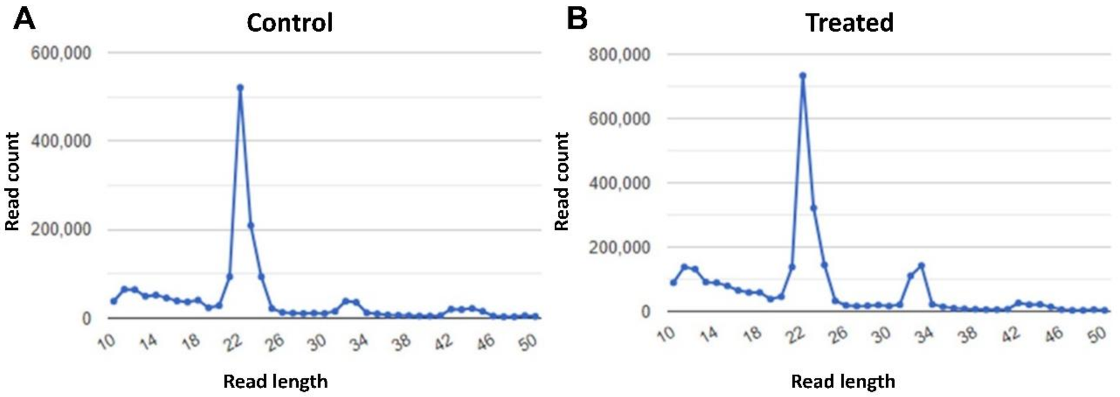

Figure S3: Profile of Small RNAs Identified by UNITAS Software. After sequencing, the filtered small RNAs, analyzed using the UNITAS software, showed higher abundance of sizes ranging from 19 to 25 nt in both control and treated group samples. A low quantity of other small RNA sizes, ranging from 10 to 50 nt, was also observed.

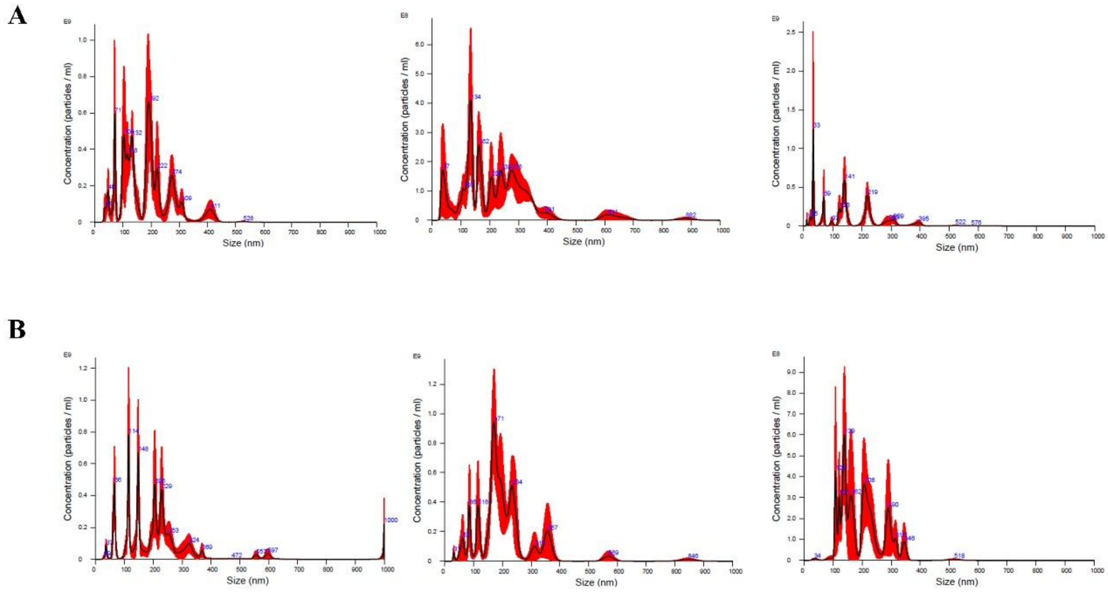

Figure S4. Nanoparticle tracking analysis (NTA) of extracellular vesicles (EVs) isolated from the conditioned medium of MDA-MB-231 cells. EVs were isolated from the supernatant of MDA-MB-231 cultures and analyzed using NTA to assess particle concentration and size distribution. (A) Particle size distribution profiles (particles per nm) for each biological replicate in the control group. (B) Particle size distribution profiles for each biological replicate in the treated group. Data represent individual replicates obtained from independent isolations.

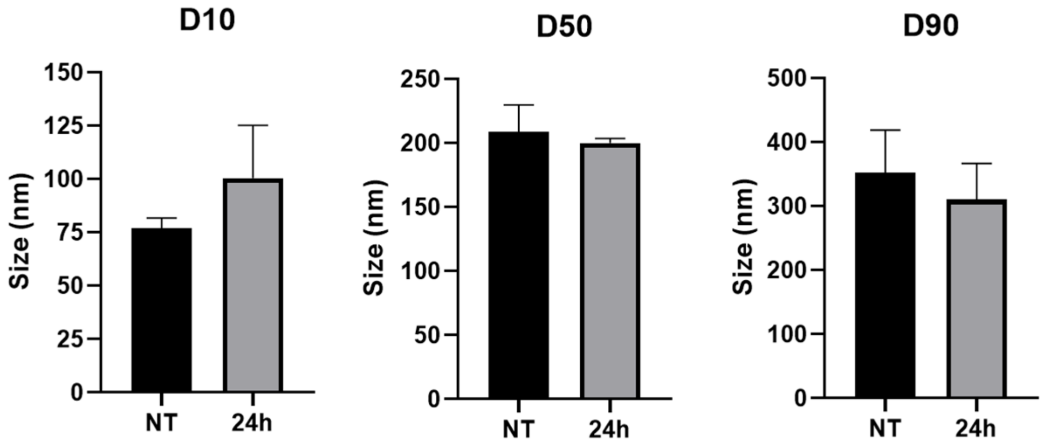

Figure S5. NTA of EVs. The graphs compare the number-based particle size distribution parameters (D10, D50, and D90, in nanometers, nm) for non-treated (NT) and after 24 hours of incubation with rhPTX3. The NTA system provides a number-based size distribution, where D10, D50, and D90 represent the diameters below which 10%, 50% (the median particle size), and 90% of the total number of quantified particles are found, respectively.

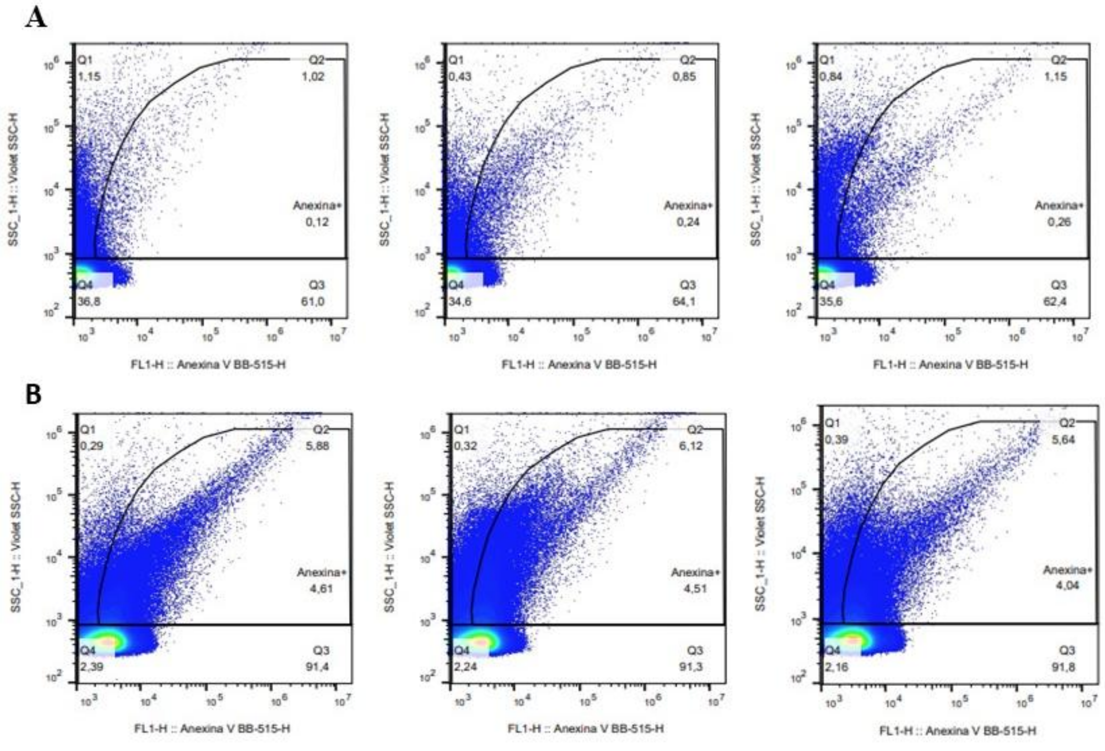

Figure S6. **Dot plots showing Annexin V positive cell populations**. Panel A: distribution of Annexin V positive cells within the defined gate for each replicate of the control group. Panel B: distribution of Annexin V positive cells within the defined gate for each replicate of the rhPTX3-treated group.
